# Identifying Training, Diagnostic and Therapeutic Needs From a Comparison in the Distribution of Vestibular Disorders in Primary Care and in a Neurotology Unit

**DOI:** 10.3389/fneur.2020.605613

**Published:** 2020-11-20

**Authors:** Emilio Domínguez-Durán, Carolina Moreno-de-Jesús, Lucía Prieto-Sánchez-de-Puerta, Irene Mármol-Szombathy, Serafín Sánchez-Gómez

**Affiliations:** Unidad de Gestión Clínica de Otorrinolaringología, Hospital Universitario Virgen Macarena, Sevilla, Spain

**Keywords:** vestibular diseases, epidemiology, health resources, primary health care, secondary care

## Abstract

**Introduction:** Several epidemiological studies in Neurotology have been previously carried out in the general population. This approach is useful for learning about the most common disorders in clinical population, but it may fail when one is trying to help professionals to guide their training, to optimize their resources and to decide on the highest-priority research objectives.

**Objective:** To identify which of the neurotological diseases are most common in two different populations, those who attended a consultation in the Neurotology Unit of a tertiary level hospital and those who did so in Primary Care in order to infer which of them requires more attention in each context and their specific needs.

**Methods:** All the diagnoses made in Hospital Care between October 15, 2017 and October 14, 2018 were reviewed. These diagnoses were coded and classified into syndromes and diseases. Later, the proportions of each category were compared with the proportions of the neurotological diagnoses made in five Primary Care centers over the same period of time.

**Results:** BPPV is the most common cause of vestibular symptoms in both contexts. Vestibular migraine, ischemic vestibular symptoms, orthostatic hypotension and side effects of drugs are common in Primary Care, whereas Ménière's disease and undifferentiated episodic vestibular syndrome are common in specialized centers.

**Conclusion:** The proportion of diagnoses in neurotologic patients is different in the general population and in the specialized center population, and therefore they have different needs. Primary Care professionals would benefit from training on maneuvers for repositioning otoliths, the treatment of headache, the identification of cardiovascular risk factors, the orthostatic hypotension and the side effects of the most commonly used drugs. The professionals who work in specialized centers need strategies for dealing with cases of BPPV associated to other vestibular diseases and refractory cases and their research should focus on the development of new diagnostic tools for the diagnosis of undifferentiated episodic vestibular syndrome and new therapeutic options for Ménière's disease.

## Introduction

In recent years, the amount of literature on the epidemiology of vestibular disorders has grown and several case series on the epidemiology of vestibular disorders have been published. These series have tried to infer how common each neurotologic disease is in their respective populations by using different methods, such as home interviews (1–3), telephone interviews (4–8), postal community questionnaires (9–12), volunteer subjects in Preventive Medicine Centers (13), general practitioners' reports (14–16) or the records of units specialized in vestibular disorders (17, 18) or other medical records (19, 20). However, the challenges of epidemiology research are not limited to finding out how common each pathology is.

One may think that all the different diseases that were detected in previous studies can be treated in the same way; however, when one examines the data more carefully, one realizes that they cannot [e.g., a single episode of benign paroxysmal positional vertigo (BPPV) that is treated early and does not relapse cannot be viewed in the same way as a severe case of Ménière's disease that does not respond well to treatment]. This is because of the amount of resources that each case will need in terms of first clinical visits, diagnostic tests, treatments and follow-up visits, as these will clearly differ. Therefore, when looking at neurotologic entities, it is not only the level of occurrence that matters, but also the impact that this entity has on both the health system and the patient's life. In this regard, while epidemiological studies that were performed in the general population showed the incidence and the prevalence of the neurotological diseases, those performed in specialized centers indicated which diseases need a greater amount of resources.

Taking into account the above, it can be assumed that professionals who work in Neurotology units and those who work in Primary Care face different situations. In relation to Neurotology units, it seems a priority that their research projects deal with diseases that cause the greatest disability in patients and diseases that consume a greater amount of resources, in order to increase the effectiveness of professionals and the development of new therapeutic tools. In relation to Primary Care, professionals should receive training to make them able to diagnose and treat the most frequent diseases and to identify patients with more serious diseases that require care in specialized units.

This study was planned in order to compare the distribution of neurotologic diagnoses between two samples of the same population: those who attended a consultation in the Neurotology Unit of a tertiary level hospital and those who did so in Primary Care. The objective of this study is to identify which of the neurotological diseases are most common in each context and to infer which of them requires more attention.

## Materials and Methods

Two samples of patients were compared in order to achieve our stated goal. The first sample was made up of a prospective series of all the patients that sought medical assistance for any type of vertigo, instability or dizziness and that belonged to the clusters of inhabitants of five voluntary Primary Care physicians (PCP) from the Public Health System. They attended either Primary Care Centers or Emergency Departments. Their symptoms needed to have started or been present between October 15, 2017 and October 14, 2018. The patients that made up this sample were diagnosed by a multidisciplinary team that included their PCPs, who were responsible for the most common diagnoses, such as BPPV, orthostatic hypotension and the side effects of frequently prescribed drugs, and also neurotologists, who were responsible for diagnosing borderline cases and making all other diagnoses. Before the start of the study, the PCPs received a 1-month training period in the Neurotology unit of the hospital. This training dealt with the most common neurotological disorders, as well as with the use of the HINTS test to diagnose stroke (21). All cases in which the PCPs had diagnostic doubts were referred to the Neurotology unit. This first sample will hereafter be referred to as the “general population.” The general population group includes all the patients of this sample, regardless of where their diagnosis was made.

The second sample was recruited retrospectively and included all the patients that visited the corresponding Neurotology Unit of the referring hospital for the patients from the first series over the same period of time. Those that had already been included in the first sample were excluded in order to avoid duplicate patients. This second sample included all the new patients that attended a consultation for the first time or that went for a follow-up consultation. This second sample will hereafter be referred to as the “specialized center population.”

Next, the age and gender of all the patients of both populations, and their diagnoses, were recorded. These diagnoses were classified using the layers I (symptoms and signs) and II (syndromes) proposed in the Overview of the International Classification of Vestibular Disorders (22) and, where possible, they were also classified using the layer III-A (disorders and diseases). Patients who did not meet the criteria for any of the disorders or diseases currently defined by the Bárány Society were diagnosed by placing them into other diagnostic categories as appropriate. Not all of the patients received a definitive diagnosis during the study period; the cut-off date for a definitive diagnosis to be made was July 17, 2020.

Later, the samples were compared in order to study the percentages of the following syndromes and diseases: acute vestibular syndromes, BPPV and other positional vertigos, episodic non-positional vestibular syndrome not attributed to ischemia, vestibular syndromes attributed to ischemia, unsteadiness and chronic vestibular syndrome (CVS), orthostatic hypotension and the side effects of medication, persistent positional-perceptual dizziness (PPPD) and miscellanea, which was made up of patients that could not be included in any of the previous groups. As some patients could have more than one diagnosis, the aforementioned groups were not mutually exclusive.

Finally, each of the aforementioned syndromes and diseases was divided, where possible, into diagnostic categories so that patients could be classified further: patients presenting with acute vestibular syndrome were divided into groups for vestibular neuritis, diseases of the central nervous system or undiagnosed diseases; patients with BPPV were divided into groups for unresolved or probable BPPV, spontaneously resolved and were also classified based on the number of affected canals; patients with episodic non-positional vestibular syndrome not attributed to ischemia were divided into groups for vestibular migraine, Ménière's disease, other less frequent or undiagnosed diseases; patients with vestibular syndromes attributed to ischemia were divided into groups for strokes and transient ischemic accidents (TIA) and, lastly, patients with unsteadiness and CVS were divided into groups for their respective etiologies where possible.

## Results

Seven hundred eighty medical records were examined, 176 were included in the general population and 604 were included in the specialized center population. There was no significant between-groups difference in the percentage of women (68.8 and 62.6%, respectively; Fisher's exact test *p* = 0.078). The age of the patients was not normally distributed, and there was a significant difference between the groups (57 and 65 years, respectively; Mann-Whitney U-test *p* < 0.001).

The results obtained from each of the syndromes and diseases studied are detailed below and the percentages are shown in Table 1:

a) Acute vestibular syndrome: seven patients in the general population group and 38 patients in the specialized center population. There was no significant difference in the percentage of patients between populations (Fisher's exact test *p* = 0.169). In the general population group, one patient was diagnosed with vestibular neuritis and the remaining patients were diagnosed as having diseases of the central nervous system; in the specialized center population, 27 patients had vestibular neuritis, five had diseases of the central nervous system and six had an acute vestibular syndromes that were not diagnosed. There was a significant difference in the distributions of patients between groups (χ^2^ test *p* < 0.001).
b) BPPV and other positional vertigos: 93 patients in the general population and 284 patients in the specialized center population were diagnosed with one of the variants of BPPV accepted by the Bárány Society (23). Sixty-three percentage of the patients in the general population were diagnosed in Primary Care. There was no significant difference in the proportion of patients diagnosed with BPPV in each of the populations studied (Fisher‘s exact test *p* = 0.101). In the BPPV group, 23.3% were diagnosed with probable BPPV, spontaneously resolved and 13.8% were diagnosed with lithiasis of multiple canals; there was no significant difference in the percentages for these subcategories between populations (Fisher's exact test *p* = 0.214 and *p* = 0.537, respectively). The number of patients presenting with positional vertigo other than BPPV was compared between groups, but no significant difference was found (Fisher's exact test *p* = 1).
c) Episodic non-positional vestibular syndrome not attributed to ischemia: 32 patients in the general population and 237 patients in the specialized center population presented with some kind of episodic, not positional vestibular syndrome other than those caused by ischemia, but the proportion was significantly higher in the latter group (Fisher's exact test *p* < 0.001). When the different subcategories were analyzed, significant differences were found in the proportions of patients diagnosed with “actual” (not probable) vestibular migraine and definite Ménière's disease (Fisher's exact test *p* < 0.001 for both), and also in the proportions of patients for whom no diagnosis could be made (Fisher's exact test *p* = 0.03).
d) Vestibular syndromes attributed to ischemia: 13 patients in the general population and 19 patients in the specialized center population were included in this group, and the difference between these groups was significant (Fisher's exact test *p* = 0.014). Twenty-five percentage of them suffered from a stroke and 75% of them suffered from one or more TIAs. There was no significant difference in the distribution of these two diagnoses between groups (Fisher's exact test *p* = 0.413).
e) Unsteadiness and chronic vestibular syndrome: 24 patients in the general population and 74 patients in the specialized center population attended a consultation because of unsteadiness; there was no statistically significant difference between the proportions in each group (Fisher's exact test *p* = 0.354). The most common cause of the symptoms in this group was cerebral small vessel disease (CSVD). There was no significant difference in the proportion of CSVD between the two groups when all the patients were considered (Fisher's exact text *p* = 0.076) or when only patients with CVS were selected (Fisher's exact test *p* = 0.060). The second most common cause of symptoms in this group was bilateral vestibulopathy, which was significantly more common in the specialized center population (Fisher's exact test *p* = 0.012). Other causes of CVS, such as uncompensated vestibular deficit, acoustic nerve neurinoma or downbeat nystagmus syndrome, were much less common and there was no significant difference in the distributions between groups (Fisher's exact test *p* = 0.241, *p* = 0.681, and *p* = 0.237, respectively). Some syndromes were so uncommon that it was impossible to compare the populations. These included two cases of cervical myelopathy, one case of mesencephalic disease, one case of myopathy and one case of mechanic unsteadiness in the lower limbs in the general population, as well as two cases of space occupying lesions of the brain, one case of idiopathic intracranial hypertension, one case of neuropathy, one case of multisystemic atrophy, one case of Chiari malformation, one case of recurrent fever associated with unsteadiness and one case of mechanic unsteadiness in the lower limbs in the specialized center population. In each group there was one case of CVS that could not be diagnosed. The aforementioned diseases are not mutually exclusive, and more than one disease was diagnosed in 13.2% of patients.
f) Orthostatic hypotension and side effects of medication: 33 patients in the general population and 25 patients in the specialized center population were diagnosed with one of these entities, and the proportion of each was significantly higher in the general population (Fisher's exact test *p* < 0.001). 69.7% of the patients in the general population were diagnosed in Primary Care. Orthostatic hypotension was found in 26 patients in the general population group and in 15 in the specialized center population, while side effects of medication were found in 9 and 12 patients respectively. Antihypertensive drugs were the drugs most commonly associated with these diseases (81.2%).
g) Persistent positional-perceptual dizziness: five patients in the general population group and 38 patients in the specialized center population were diagnosed with PPPD; no significant between-groups difference in the proportion of patients was found (Fisher's exact test *p* = 0.051). 30.2% of these patients were considered primary cases, whereas in the remaining 69.8% they were seen as being secondary to another preexisting neurotologic condition (confidence interval 95% 53.7–82.3%). There was no significant difference in the distribution of primary and secondary cases between the populations (Fisher's exact text *p* = 0.518). The triggers of the 30 secondary cases were BPPV (13 cases), vestibular neuritis (four cases), stroke (one case), TIAs (two cases), vestibular migraine (15 cases), Ménière's disease (one case), myelopathy (one case), CSVD (four cases), uncompensated vestibular deficit (one case) or side effects of medicaments (two cases). The previous triggers are not mutually exclusive and therefore their sum is higher than 30.
h) Miscellanea: 32 patients could not be included in any of the previous categories. These included seven patients with presyncopal dizziness, four patients with altered eye movement, three patients with panic attacks, one patient with anemia, one patient with hyperthyroidism and one patient with myasthenia gravis. In five patients, no diagnosis could be made, and nine patients failed to attend their medical appointment. There was a significant difference in the proportion of the miscellanea group between the populations (Fisher's exact test *p* = 0.037) and this was attributed to the different ways in which missing patients were measured in the two populations.

**Table 1:** Between-groups comparison of the percentage of different diseases.

**Diagnostic syndrome**	**Subcategories**	**General population group (176)**	**Specialized center group (604)**
Acute vestibular syndrome		4.0%	6.3%
	Vestibular neuritis^*^	14.3%	71.1%
	Central nervous system^*^	85.7%	13.2%
	Undiagnosed	0%	15.8%
BPPV		52.8%	47.0%
	Probable BPPV resolved spontaneously	26.9%	22.2%
	Lithiasis of multiple canals	14.0%	13.7%
Positional vertigo different from BPPV		0.6%	1.2%
Episodic non-positional vestibular syndrome not attributed to ischemia^*^		18.2%	39.2%
	Vestibular migraine^*^	71.9%	32.9%
	Probable vestibular migraine	28.1%	13.9%
	Ménière's disease^*^	0%	31.6%
	Probable Ménière's disease	0%	4.6%
	Other diagnosed causes	0%	5.2%
	Other undiagnosed causes^*^	0%	11.8%
Vestibular syndrome attributed to ischemia^*^		7.4%	3.1%
	Stroke	30.8%	21.1%
	Transient ischemic attack	69.2%	78.9%
Unsteadiness and chronic vestibular syndrome		13.6%	12.3%
	Cerebral small vessel disease	70.8%	50.5%
	Bilateral vestibulopathy^*^	4.2%	27.0%
	Uncompensated unilateral vestibular deficit	4.2%	12.2%
	Acoustic nerve neurinoma	4.2%	4.1%
	Downbeat nystagmus syndrome	0.0%	6.8%
Orthostatic hypotension and side effects of medication^*^		18.8%	4.1%
	Orthostatic hypotension	78.8%	60.6%
	Side effects of medication	27.3%	48.0%
PPPD		2.8%	6.3%
Miscellanea^*^		6.8%	3.3%

The total number of individuals in each group is indicated in brackets. The percentage indicated for each of the subcategories is calculated from the total of patients in each syndrome. The pathologies marked with “

* *” were those in which significant between-groups differences were found. The subcategories in the group unsteadiness and CVS and in the group orthostatic hypotension and side-effects of medication were not collectively exhaustive and in the case of unsteadiness and CVS they were not mutually exclusive either*.

## Discussion

Our results indicate that the populations that present otoneurological symptoms in Primary Care and in Hospital Care are different; at least in our health system. Therefore, in our area, the resources allocated to each area must be different too, and the training given to professionals and the most relevant lines of research must also be specific to that healthcare context. Caution should be exercised when extrapolating our results to other health systems because different relationships between PCPs and neurotology specialists could change the results obtained. Furthermore, an analysis of the subcategories within each diagnostic group has led the authors to propose the following hypotheses:

### Vestibular Neuritis: Uncommon but Very Noticeable?

Although the percentage of acute vestibular syndromes can be considered to be similar in both groups, it is interesting to note that diseases of the central nervous system were more common in the general population group. Traditionally, it has been argued that the most common cause of acute vestibular syndrome is vestibular neuritis, but this is not corroborated by our results. On the one hand, this could be due to the fact that the previous series came from specialized centers (24) and emergency departments (25) or it could also be due to the fact that magnetic resonance imaging has a low sensitivity when detecting lesions in the posterior fossa (26), thus leading to the underdiagnosis of small strokes. On the other hand, vestibular neuritis may have been less common in the general population because it is accompanied by an abrupt and intense crisis of vertigo which may mean that patients tend to seek medical attention in hospitals, whereas central nervous system vertigo symptoms can be more subtle, and this might be why they are more common in Primary Care centers. In any case, our results suggest that vestibular neuritis is not as common as was once thought. It is also important to note that no diagnosis could be made in a considerable number of cases (15.8%) in the specialized center population. This is similar to data collected by Yebra-González et al. (27), where 39.2% of acute vertigo was of unclear origin.

### Two Different Benign Paroxysmal Positional Vertigos

If one considers the data obtained for BPPV, one realizes that it was the most common disorder in both populations, but one may also wonder why the incidence of BPPV was similar in both groups. We attribute this to the fact that there could have been less cases associated to other neurotologic diseases in the general population group and more cases associated to other neurotologic diseases and refractory to treatment cases in the specialized center group. If the proportion of cases non-associated to other neurotologic diseases was similar in both contexts, then the percentage of spontaneously resolved cases should have been more different. This hypothesis was based on the fact that in the general population group the time required to get a consultation with a general practitioner is 1 week, whereas in the specialized center group this can take up to 4 months. Thus, if one considers the data obtained by Álvarez-Morujo et al. in which they estimate that the percentage of patients whose BPPV spontaneously resolves increases with time (28), the percentage of spontaneously resolved cases in the specialized center should have been significantly higher. A further analysis of this data also showed that the percentage of cases of BPPV associated with acute and episodic vestibular syndromes was significantly higher in the specialized center population (20.1 vs. 10.8%, Fisher's exact test *p* = 0.026), thus supporting this hypothesis. Based on our experience and taking into account that the cases of BPPV treated in Primary Care are simpler, PCPs could benefit from a training in otolith repositioning maneuvers. None of the volunteer PCPs who carried out this study performed these maneuvers before their training in the Neurotology unit; After training, they were able to identify the symptoms of the disease more often, treat the simplest cases, and refer doubtful cases.

### Ménière's Disease: Still a Needle in the Haystack

Episodic non-positional vestibular syndrome not attributed to ischemia was more common in the specialized center population than in the general population. A closer look at this group showed that this could be due to the presence of patients diagnosed with Ménière's disease in the specialized center population that were not present in the general population group. This phenomenon was described quite a while ago and it was called “the needle in the haystack” (7). Based on a comparison of our groups, we can conclude that professionals who deal with neurotologic patients in Primary Care would benefit from a better knowledge of how to treat patients suffering from vertigo and headache (16), whereas specialized centers should handle the diagnosis and management of patients suffering from Ménière's disease. Headache treatment is a basic competence of the PCP. Even if the PCP is not prepared to make the diagnosis of vestibular migraine, knowing the association between headache and vestibular symptoms can help improve the patient's symptoms by prescribing analgesic treatments while requesting an evaluation in a specialized unit. It is important to note that a considerable number of patients in the specialized center population could not be diagnosed, and this was due to difficulties when deciding whether a patient was suffering from vestibular migraine or Ménière's disease. Fortunately, new diagnostic tools that will help to differentiate between these two conditions, such a genetic tests or detection of inflammatory citoquines, are being developed so presumably some of these patients will be able to be classified in the near future (29).

### Mild Neurotologic Symptoms Due to Ischemia Are Underdiagnosed

Our Neurotology Unit is the reference unit for a population that is 60 times bigger than the general population sampled for this study; however, the number of patients found to be suffering from ischemic vestibular syndromes was not even two times higher in absolute numbers. This may be due to the fact that when a stroke was diagnosed in an Emergency Room, the patient was sent to the stroke unit, instead of the Neurotology Unit. However, making a diagnosis of TIA accompanied by vestibular symptoms is much more difficult and quite a lot of the patients that suffered from them should have been referred to the Neurootology Unit. This seems to support the hypothesis that ischemic vestibular syndromes are underdiagnosed in our population and therefore more training about the relationship between cardiovascular risk and vestibular symptoms is needed in Primary Care and Emergency Rooms. Based on our own experience, it is possible to train PCPs in the use of the HINTS test. Although this test is not easy to perform, we noted that the training increased the awareness of the PCPs in the possibility of an ischemic etiology in many cases. We believe that it is preferable that PCPs refer doubtful cases of HINTS test and these are evaluated by experienced professionals to confirm the diagnosis.

### When Is Cerebral Small Vessel Disease a “Incidental” Finding?

The most common cause of CVS symptoms in both populations was CSVD, which was significantly more common than unilateral or bilateral vestibular deficits (Z-test for a comparison of the column proportion with *p*-values adjusted using the Bonferroni method, considering patients suffering from cerebral small vessel disease as the first group, and patients with unilateral or bilateral vestibular deficit as the second group and patients with both as the third group *p* < 0.05). CSVD is diagnosed using magnetic resonance imaging. It is often found in the imaging of asymptomatic subjects and affects almost everyone over the age of 90 (30). Because of this, not all cases of CSVD can be assumed to be the cause of vestibular symptoms and it should only be linked to some cases of gait disturbance or recurrent strokes (30), and in the case of strokes this is only when they affect the central nervous system areas of the vestibular system. Although CSVD was the most common cause of CVS, this does not reduce the importance of the tests that study the function of the peripheral vestibular system, especially considering that vestibular deficits were the second most common cause of symptoms in this group, but it does indicate that it is necessary to evaluate the vestibular system as a whole and not as a sum of separate organs. Sometimes, as we found in both populations, CVS is not the result of just one lesion, but rather the sum of many.

### Antihypertensive Drugs and Vestibular Symptoms

When this study was planned, orthostatic hypotension and the side effects of medication were considered as separate conditions. However, after analyzing the results, we discovered that antihypertensive drugs played an important role in both diseases and thus they were grouped together. Orthostatic hypotension and the side effects of medication were statistically more common in the general population group. We attribute this to the fact that these are entities that are easy to recognize; sometimes, the association between drugs and symptoms is reported by the patient themselves. In our study, PCPs were able to differentiate between orthostatic symptoms and BPPV easily, but this fact could be independent from the received training. This may explain they may be less common in the specialized center population.

### The Rise of Persistent Positional-Perceptual Dizziness

Finally, we can apply the same reasoning that was used for the ischemic vestibular syndromes to PPPD: if the specialized center serves a population that is 60 times greater than the sample of the general population, a larger absolute number of subjects with this diagnosis should have been found. PPPD should be the second most common diagnosis in specialized centers (31) and this diagnosis is not easy as it is necessary to systematically rule out other diseases whose symptoms are quite similar. PPPD is a term that has been recently described, and because of this professionals need to take this diagnostic option into account more often. However, the diagnosis of PPPD can become a double-edged sword: although it is incredibly useful for the diagnosis of patients whose symptoms could not be explained by reference to other older and better-known diseases, we are afraid that in many patients in which PPPD is a part of the path to recovery this label could complicate the diagnostic process.

## Conclusion

The proportion of diagnoses in patients with vestibular symptoms is different in the general population and in the specialized center population, and therefore the two contexts have very different diagnostic and therapeutic needs and require specialized training for their staff. BPPV is the most common cause of vestibular symptoms in both contexts, but while Primary Care professionals would benefit from training on maneuvers for repositioning otoliths, those working in specialized centers need strategies for dealing with cases associated to other vestibular diseases and refractory cases. Also, further research and training is required into the treatment of headache and cardiovascular risk factors and the identification of orthostatic hypotension and the side effects of the most commonly used drugs in patients with vestibular symptoms in Primary Care; whereas specialized centers should focus on the development of new diagnostic tools for the diagnosis of undifferentiated episodic vestibular syndrome and new therapeutic options for Ménière's disease.

## Data Availability Statement

The raw data supporting the conclusions of this article will be made available by the authors, without undue reservation.

## Ethics Statement

The studies involving human participants were reviewed and approved by the Ethics Committee of the University Hospitals Virgen Macarena and Virgen del Rocío, Sevilla, Spain. Written informed consent for participation was not required for this study in accordance with the national legislation and the institutional requirements.

## Author Contributions

ED-D and IM-S contributed in the diagnoses of the patients of the series. CM-d-J and LP-S-d-P recorded and analyzed the database for the study. SS-G contributed in the diagnoses of the patients of the series and directed the project research. All authors contributed to the article and approved the submitted version.

## Conflict of Interest

The authors declare that the research was conducted in the absence of any commercial or financial relationships that could be construed as a potential conflict of interest.
